# The Effect of a Family-Based Lifestyle Education Program on Dietary Habits, Hepatic Fat and Adiposity Markers in 8–12-Year-Old Children with Overweight/Obesity

**DOI:** 10.3390/nu12051443

**Published:** 2020-05-16

**Authors:** Lide Arenaza, María Medrano, Maddi Oses, Maria Amasene, Ignacio Díez, Beatriz Rodríguez-Vigil, Idoia Labayen

**Affiliations:** 1 Institute for Innovation and Sustainable Development in Food Chain (IS-FOOD), Public University of Navarra, Calle Tajonar 22, 31006 Pamplona, Navarra, Spain; maria.medrano@unavarra.es (M.M.); maddi.oses@unavarra.es (M.O.); idoia.labayen@unavarra.es (I.L.); 2 Navarra’s Health Research Institute (IdiSNA), 31008 Pamplona, Navarra, Spain; 3 Department of Pharmacy and Food Sciences, University of the Basque Country, UPV/EHU, 01006 Vitoria-Gasteiz, Araba, Spain; maria.amasene@ehu.eus; 4 Pediatric Endocrinology Unit, University Hospital of Araba (HUA), 01009 Vitoria-Gasteiz, Spain; IGNACIO.DIEZLOPEZ@osakidetza.eus; 5 Department of Magnetic Resonance Imaging, Osatek, University Hospital of Araba (HUA), 01009 Vitoria-Gasteiz, Spain; brodriguezvigil@osatek.eus

**Keywords:** lifestyle program, dietary habits, hepatic fat, sugar-sweetened beverages

## Abstract

Healthy lifestyle education programs are recommended for obesity prevention and treatment. However, there is no previous information on the effects of these programs on the reduction of hepatic fat percentage. The aims were (i) to examine the effectiveness of a 22-week family-based lifestyle education program on dietary habits, and (ii) to explore the associations of changes in dietary intake with percent hepatic fat reduction and adiposity in children with overweight/obesity. A total of 81 children with overweight/obesity (aged 10.6 ± 1.1 years, 53.1% girls) and their parents attended a 22-week family based healthy lifestyle and psychoeducational program accompanied with (intensive group) or without (control) an exercise program. Hepatic fat (magnetic resonance imaging), adiposity (dual energy X-ray absorptiometry) and dietary habits (two non-consecutive 24 h-recalls) were assessed before and after the intervention. Energy (*p* < 0.01) fat (*p* < 0.01) and added sugar (*p* < 0.03) intake were significantly reduced in both groups at the end of the program, while, in addition, carbohydrates intake (*p* < 0.04) was reduced exclusively in the control group, and simple sugar (*p* < 0.05) and cholesterol (*p* < 0.03) intake was reduced in the exercise group. Fruit (*p* < 0.03) and low-fat/skimmed dairy consumption (*p* < 0.02), the adherence to the Mediterranean Diet Quality Index for children and teenagers (KIDMED, *p* < 0.01) and breakfast quality index (*p* < 0.03) were significantly higher in both control and intervention groups after the intervention. Moreover, participants in the exercise group increased the adherence to the Dietary Approaches to Stop Hypertension (DASH) diet (*p* < 0.001), whereas the ratio of evening-morning energy intake was significantly lower exclusively in the control group after the program (*p* < 0.02). Changes in energy intake were significantly associated with changes in fat mass index (FMI) in the exercise group, whereas changes in sugar-sweetened beverages (SSB) consumption was associated with percent hepatic fat reduction (*p* < 0.05) in the control group. A 22-week family-based healthy lifestyle program seems to be effective on improving diet quality and health in children with overweight/obesity and these should focus on SSB avoidance and physical activity.

## 1. Introduction

The worldwide rising prevalence of childhood obesity is of public health concern [1]. Childhood obesity is associated with greater cardiometabolic risk later in life [2], and already in early ages [3]. Spain, with 26% and 12.6% of 8–17 years-old children presenting overweight and obesity, respectively, is one of the European countries with the highest prevalence of childhood obesity, reporting a worse situation in 8–13 years-old boys from low socioeconomic and educational families [4]. National strategies, where environmental and policy actions are a priority, have been launched to tackle pediatric obesity in this country [5]. Abdominal adiposity is strongly associated with non-alcoholic fatty liver disease (NAFLD) [6], which consists of the accumulation of triglycerides within hepatocytes with the absence of alcohol consumption [7]. NAFLD is also considered as the hepatic manifestation of metabolic syndrome [8].

Lifestyle changes are the main recommendation for the prevention and treatment of NAFLD. Diet quality improvement, with a special focus on sugar-sweetened beverage (SSB) avoidance, and increasing physical activity together with reducing sedentary behaviors, are recommended as the main targets to improve the spectrum of the disease [9]. Besides the importance of an early treatment of obesity and NAFLD in order to prevent adiposity-related comorbidities, lifestyle changes seem to be more effective in childhood than later life, since children are still acquiring and establishing their habits. Furthermore, the participation and active involvement of the parents in lifestyle interventions increase their effectiveness, since they are fundamental agents in their children’s lifestyle habits [10,11,12]. As far as we are aware, there is no previous information on the effects of lifestyle education programs targeting diet quality improvement on the reduction of hepatic fat percent in prepuberal children. In adults and adolescents with obesity, lifestyle intervention based on weight loss induced by hypocaloric diets have been shown to be effective at reducing hepatic fat [13,14,15] or were based on exercise programs [16]. However, caloric restriction is not recommended in the pediatric population since it could affect both children’s growth and development, as well as induce eating disorder behaviors development [17].

Therefore, the aims of the current study were (i) to examine the effects of a family-based lifestyle educational program on dietary habits and (ii) to explore the associations of changes in dietary habits with changes in percent hepatic fat and adiposity in children with overweight/obesity.

## 2. Material and Methods

### 2.1. Study Design and Participants

**Figure 1:**
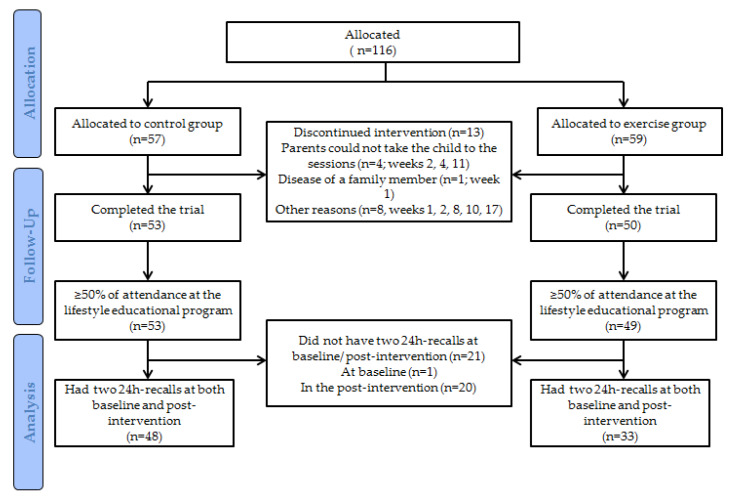
Flowchart of children participating in the study.

The EFIGRO study is a non-randomized two-arm, parallel-design controlled trial (NCT02258126, ClinicalTrials.gov), carried out between 2014 and 2017 in Vitoria-Gasteiz (Spain). The study protocol was approved by the Ethic Committee of Clinical Investigation of Euskadi (PI2014045) and was performed according to the compliance of the ethical guidelines of the Declaration of Helsinki. More detailed information and the main effects of the study have been published elsewhere [18,19]. Briefly, participants were divided into control and exercise groups, in which the main difference was that whereas children in the control group participated exclusively in the family-based healthy lifestyle program, the ones in the exercise group besides participating in the previous one additionally did in the exercise program (3 sessions per week). For the current purpose, a total of 81 children with overweight/obesity (aged 10.6 ± 1.1 years, 53.1% girls) either from the control or exercise group who (1) completed the study, (2) attended at least 50% of the lifestyle education sessions, and (3) had two 24 h-recalls at baseline and post-intervention, were included in the analyses (Figure 1).

### 2.2. Family-based Healthy Lifestyle Program

All children and their parents or caregivers participated in a 22-week intervention focused on healthy lifestyle promotion. The program, conducted by experienced nutritionists, was composed of 11 sessions (once per two weeks), whose main aim was to improve dietary habits by increasing the intake of fruits and vegetables (F+V), enhancing breakfast habits, and reducing sugar intake and SSB consumption, as well as to make children be physically more active. Children had a booklet which included the objectives to accomplish from each session and these were commented on thereafter. In order to make easier to adopt the proposed lifestyle changes, both children and parents also attended psychoeducational sessions to achieve appropriate skills and counseling. More details about the interventions has been published elsewhere [18].

### 2.3. Hepatic Fat and Adiposity

Body weight (SECA 760), height (SECA 220) and waist circumference (SECA 201) were measured following standard protocols and BMI was calculated. Children were classified as overweight/obese according to the World Obesity Federation criteria [20]. Dual energy X-ray absorptiometry (DXA; HOLOGIC, QDR 4500 W) was used to measure total and abdominal adiposity [18]. Fat mass index (FMI) was calculated as fat mass divided by height squared (kg/m^2^). Liver fat percentage was measured by magnetic resonance imaging (MAGNETOM Avanto 1.5-T, Siemens, Munich, Germany) with a Dixon method.

### 2.4. Dietary Habits

#### 2.4.1. Dietary Intake

Energy and nutrients intake was examined using two non-consecutive 24 h-recalls by trained dietitians and nutritional composition was analyzed with the EasyDiet software (© Biocentury, S.L.U. 2020, Spain). Thereafter, the average intake of the two reported days was calculated. We also calculated added sugar intake by subtracting natural sugar from fruits and dairy products from total simple sugar intake.

#### 2.4.2. Adherence to Dietary Patterns

Adherence to dietary patterns was examined, both before and after the participation in the trial. More information about the tools and criteria used to examine the adherence to these patterns or scores is detailed in Table S1. Mediterranean Diet Quality Index for children and teenagers (KIDMED test) was used to examine the adherence to the Mediterranean pattern [21]; the adherence to the Dietary Approaches to Stop Hypertension (DASH) diet was assessed by following the Cohen et al. criteria [22] and HDI score by using World Health Organization (WHO) criteria [23].

#### 2.4.3. Breakfast Habits

Skipping breakfast prevalence was obtained from the food recalls, and the breakfast quality index (BQI) was used to examine the children’s breakfast quality before and after participating in the education program [24]. This information is available in the supplementary material (Table S1).

#### 2.4.4. Meal Frequency and Daily Energy Distribution (Morning vs. Evening Energy Intake)

Eating frequency was obtained from the 24 h-recalls and each eating occasion was considered when meeting the criteria of (1) having ≥50 kcal/meal, and (2) spending ≥15 min from any previous eating occasion as described elsewhere [25]. The average of daily energy intake was divided into morning and evening intake. The criteria used in other studies for morning and evening classification has been before and after 12 p.m., respectively [26]. Nevertheless, taking into consideration Spanish dietary habits, in which none of the participants used to eat lunch before noon, we used another criterion. Thereby, evening/morning ratio (EME_ratio_) was calculated as evening energy intake divided by morning energy intake establishing as threshold before and after having lunch, by considering the intake of lunch as morning intake.

### 2.5. Statistical Analysis

Independent *t* tests were used to analyze baseline differences according to intervention groups for continuous variables, whereas chi-square test was used for categorical variables. To examine changes in nutritional variables between baseline and post intervention, paired sample *t*-test and McNemar test were used for continuous and categorical variables, respectively. Changes in biological and nutritional variables were calculated as post-value subtracted by pre-value (Δ = post-pre). Univariate linear models (ANCOVA) were performed to examine differences in changes in continuous variables (dependent variables) using study group as fixed factor and sex, age and changes in energy as covariates. Partial correlations were performed to examine the association between changes in dietary intake and changes in hepatic fat and adiposity, adjusting for sex and age and changes in height and energy intake. The interaction effect of sex and intervention group (control or exercise) was examined with repeated measures ANCOVA, by using pre- and post-intervention energy and macronutrients intake, as within subjects’ variables. Statistical analyses were carried out with the statistical software SPSS version 20.0 (SPSS Inc., Chicago, IL, USA), with the significance level of α = 0.05.

## 3. Results

Table 1 shows baseline characteristics of participants according to intervention group. There were no significant baseline differences between the two intervention groups in biological and sociodemographic characteristics or body composition measurements. Intervention effects on BMI and percent hepatic fat (*p*s < 0.01) were significantly different according to the study group, with greater effects on participants in the exercise group, as has been published elsewhere [19].

### 3.1. Dietary Intake

Table 2 shows baseline differences in dietary habits between the control and exercise group, changes in dietary habits at the end of the intervention in both groups (post- vs. pre-intervention values), as well as differences in the effect of the intervention on dietary habits between control and exercise groups. It can be observed that there were no baseline differences in dietary habits between the two intervention groups (*p* > 0.05, Table 2).

It was observed that both groups significantly reduced energy and fat intake, as well as added sugar intake after participating in the program (*p* < 0.05, Table 2). Similarly, simple sugar intake was also reduced at the end of intervention in both groups, although not significantly in the control group (*p* < 0.07). Regarding food groups, fruits and low-fat/skimmed dairy consumption were significantly increased in both control and intervention groups (*p* < 0.03). Moreover, the adherence to dietary patterns was also improved regardless of the study group, as KIDMED and BQI score were significantly increased after participation in the program (*p* < 0.03). There were no significant differences between the intervention groups.

Carbohydrates intake and EME_ratio_ was significantly reduced exclusively in the control group (*p* < 0.04), whereas cholesterol intake was significantly reduced, and the DASH score increased in the exercise group, but not in the control one (*p* < 0.03). However, no statistically significant differences were found between groups in the previous variables. Although changes in cereal intake were significantly different between groups (*p* < 0.03), none of the groups showed a significant intervention effect in its intake (*p* > 0.05). A significant increase in the DASH score was observed exclusively in the exercise group (*p* < 0.001), showing a significant intervention effect between groups (*p* < 0.05).

### 3.2. Association between Dietary Improvements and Changes in Hepatic Fat and Adiposity Markers

Associations of changes in dietary intake, particularly focused on those items related to the main nutritional goals of the program, and dietary patterns with hepatic fat and adiposity markers are shown in Table 3 and separately by intervention group in Table S2. Changes in SSB consumption was significantly associated with changes in hepatic fat content (*p* < 0.05), but not with changes in FMI or abdominal fat. When the results were examined by intervention group, it was observed that the association between changes in SSB consumption and changes in percent hepatic remained statistically significant only in the control group (*p* < 0.05), as shown in Table S2. Moreover, changes in energy intake were significantly associated with changes in FMI only in the exercise group (*p* < 0.05, Table S2). No other significant associations were found between dietary improvements and changes in percent hepatic fat or adiposity markers. In addition, Figure S1 provides data on children’s growth over the study time.

## 4. Discussion

The current study showed interesting findings regarding the effect of a 22-week family-based healthy lifestyle intervention program on changes in dietary habits and adiposity markers in children with overweight/obesity. Firstly, our intervention program was effective on reducing the intakes of energy, carbohydrate, total fat and simple and added sugar intake, as well as increasing the consumption of fruits and low-fat/skimmed dairy in children with overweight/obesity. Consequently, the adherence to the Mediterranean and DASH dietary patterns improved, together with an enhancement in the breakfast quality and daily energy distribution after participating in the program. Secondly, changes in SSB consumption were associated with hepatic fat reductions, even in the absence of exercise training.

Overall, children had poor dietary habits, with only 16% of children showing optimal Mediterranean diet adherence (KIDMED score ≥8 points) at baseline. Della Corte et al. [27] reported similar results in children and adolescents with obesity; only 14.8% of participants had an optimal KIDMED score. Concerning macronutrients, the average fat percentage intake in our study sample (41%) exceeded the one established for the Mediterranean diet (30–35%). The intake of percent added sugar (11%) was also above WHO recommendations (<10% of total energy intake, with additional health benefits if <5%) [28].

After the program, children improved their dietary habits. One of the main goals of the program was successfully achieved, as participants had lower intakes of simple and added sugar after the intervention. Moreover, total fat intake decreased among participants after the intervention, while protein intake barely changed. Due to lower carbohydrate and fat intake, despite not being the main goal of the program, the intervention induced an average reduction of ~200 kilocalories/day after the study, which might be due to healthier and less caloric dietary choices. Similarly, Reinehr et al. [29] reported reductions in energy, fat and sugar intake after a 6-month intervention based on nutritional education, behavior counselling and physical activity in 8–16 years-old overweight children.

Regarding food groups, the aim of promoting F+V consumption was partially accomplished. F+V consumption was increased in 52% and 24%, respectively, at the end of the intervention, although the latter was not significant. F+V consumption is highly recommended for all age-groups, not only because of their low caloric content, but also due to their fiber and micronutrients supply; therefore, their intake is essential for obesity prevention and treatment [30,31]. Despite the increase of vegetable consumption, participants still consumed fewer amounts than that which was recommended. In fact, after the participation in our program, the average intake of F+V among children was 332 g/day, not meeting the recommendations of the American Heart Association (>360 g/day) [32] or the WHO guidelines (>400 g/day) [33]. This is in line with a previous work conducted in the same region of our study (Basque Country) among 4–18 years-olds [34]. We included cooking workshops in two educational sessions in order to increase kids’ interest and motivation regarding healthy food preparation and to make them feel involved throughout the whole process. Nonetheless, it might be interesting to additionally include F+V tasting workshops in these type of programs, since they have been to succeed in enhancing youth’s interest in healthy eating [35,36].

With respect to dairy intake, low-fat/skimmed dairy consumption increased by 73.5% after the intervention, as participants tended to choose milk/yoghurts with less fat content than they used to. Nevertheless, participants still did not meet the recommendations for dairy products (500 g/day) [37]. Although these products are an appropriate source of calcium, this mineral intake was below European Food Safety Authority recommendation [38] (800–1150 mg/day for 4–17 years-olds), even after the program.

SSB consumption was not significantly changed after the program, even though the 43% reduction in its consumption should be highlighted. Another 10-week intervention in 4–10 years old children based on family meals did not find intervention effects on SSB consumption [39]. Likewise, Haerens et al. [40] found no positive effects on the consumption of SSB in 11–15 years olds after a school-based program. In contrast, another combined school/community-based water campaign intervention was successful in reducing the average SSB consumption in children [41].

The adherence to Mediterranean and DASH dietary patterns was improved after the lifestyle program. Our findings are in accordance with another study [42], reporting a 42% increase in the aforementioned score in children with abdominal obesity after 8-weeks; nonetheless, this intervention included an established energy-restricted diet. As a result of the increase in F+V and low-fat dairy consumption, the intake of micronutrients included in the DASH score, such as potassium and calcium, increased, and in turn the DASH score also did. As far as we are aware, there is no other study examining changes in the adherence to DASH and HDI dietary patterns in obese children.

Skipping breakfast was less common after the program, as its frequency reduced by 62.5%. Moreover, the greater BQI score observed after the program showed an improvement in the quality of the breakfast. Despite no changes in meal frequency, participants decreased the EME_ratio_, showing that the energy intake in the morning was higher compared to the afternoon after the intervention. According to Aljuraiban et al. [38], British and American adults with lower EME_ratio_ and more eating frequents showed lower BMI and higher nutrient density, whereas those who ate less frequently and mostly in the evening showed lower intakes of F+V and lower nutrient density and higher alcohol consumption.

Concerning intervention effects on dietary habits between groups, children in the exercise group increased their cereal intake significantly, as well as their DASH score, compared to their peers in the control group. In this line, Manz et al. [43] reported a positive association of physical exercise and recommended daily physical activity with cereal intake among young boys, which suggest that nutritional adaptation as changes in food choices may occur as a response to physical activity [44]. According to the literature [45], a 15-week exercise training could help young adults pursuing healthier dietary preferences, as well as regulating their food intake in young adults. This could explain the between groups difference in DASH score, in which participants in the exercise group improved significantly more their diet quality according to DASH guidelines.

Our results pointed out a positive association between changes in energy intake and changes in FMI in the exercise group. In accordance with this, Steinsbekk et al. [46] reported that changes in energy intake from baseline to six months predicted a decrease in body fat in children with obesity. The exclusive association observed between the reduction of energy intake and the decrease in FMI in the exercise group may be explained by the additional energy expenditure due to exercise training, which would affect both energy balance and adiposity [47,48].

Changes in SSB consumption were associated with changes in percent hepatic fat. This finding is in line with our previous work [49], reporting a positive association between SSB consumption and percent hepatic fat in children with overweight/obesity, which in turn supports the evidence linking sugary drinks and liver fat [50,51]. Physical activity exerts an important effect on reducing liver fat [19], which may explain the lack of association between changes in SSB and percentage hepatic fat in the exercise group, by covering somehow the effect of SSB. However, even with the absence of exercise, the reduction of SSB consumption helps to reduce hepatic fat content. A recent systematic review confirmed the link between SSB consumption and weight gain in both children and adults [52]. Notwithstanding the number of lifestyle interventions in children with obesity, to the best of our knowledge, there are no studies targeting SSB reduction for the treatment of NAFLD in children. Findings from another study [53] suggested that a conventional low-fat or low-glycemic load diet could produce substantial decreases in liver fat and hepatocellular injury within six months in obese children.

## 5. Strengths and Limitations

This study has some strengths and limitations. The sample size could be considered as a study limitation, since it was lower than the original sample due to some missing data, especially in the post-intervention. However, our well characterized and homogeneous sample with a narrow range of age should be considered as study strength. Last but not least, the use of magnetic resonance imaging for hepatic fat determination and the study design strengthen these findings.

## 6. Conclusions

In conclusion, a 22-week family-based healthy lifestyle program mostly focused on nutritional education and accompanied by psychoeducation seemed to be effective in improving dietary habits in children with overweight/obesity by reducing energy intake; total carbohydrate, simple and added sugar intake; and total fat intake on the one hand, and by increasing the consumption of fruits and low-fat/skimmed dairy on the other. Furthermore, these changes resulted in improvements in the adherence to the Mediterranean and DASH dietary patterns, as well as in the breakfast quality and daily energy distribution after the program. The association observed between SSB consumption and hepatic fat reduction in participants who did not additionally attend the exercise program support the detrimental effects of sugary drinks on cardiometabolic health. Hence, interventions targeting healthy dietary and lifestyle habits with a special focus on the SSB avoidance and physical activity should be promoted to prevent fatty liver and cardiometabolic disorders in children with overweight/obesity.

## Figures and Tables

**Table 1:** Baseline biological and sociodemographic characteristics of study participants according to the intervention group.

	Control Group		Exercise Group		*p*
	N		N		
Age (years)	48	10.6 (1.1)	33	10.5 (1.1)	0.597
Girls (N, %)	48	26, 54.2	33	17, 51.5	0.814
High educational level of the mother (N, %)	48	39, 81.3	32	21, 63.6	0.114
Children with Spanish-origin mother (N, %)	48	43, 89.6	33	30, 90.9	0.844
Body mass index (kg/m^2^)	48	25.2 (2.8)	33	25.7 (3.6)	0.536
Children with obesity (N, %)	48	26, 54.2	33	19, 58	0.762
Fat mass index (kg/m^2^)	48	9.8 (2.2)	32	10.4 (2.6)	0.270
Abdominal fat (kg)	48	2.4 (1.0)	32	2.6 (1.1)	0.448
Hepatic fat (%)	48	5.1 (2.8)	33	5.8 (5.2)	0.471

Values are means (standard deviation) or frequencies and percentages (N, %).

**Table 2:** Dietary habits of children participating in the family-based lifestyle program (control group) and in the same plus exercise program (exercise group), before (Pre) and after (Post) the intervention.

	Control Group				Exercise Group				Baseline Control vs. Exercise	Intervention Effect Control vs. Exercise
	N	Pre	Post	*p* *	N	Pre	Post	*p* *	p **	p ***
**Energy, nutrients and food groups**										
Energy intake (kcal/day)	48	1827 (423)	1652 (376)	**0.009**	33	1855 (430)	1622 (326)	**0.003**	0.774	0.566
Carbohydrates intake (g/day)	48	197 (53)	179 (44)	**0.039**	33	189 (53)	178 (39)	0.248	0.501	0.168
Simple sugar intake (g/day)	48	87 (32)	78 (24)	0.067	33	84 (28)	73 (25)	**0.044**	0.622	0.970
Added sugar intake (g/day)	48	55 (31)	43 (19)	**0.024**	33	58 (29)	40 (19)	**0.006**	0.955	0.517
Fat intake (g/day)	48	81 (28)	70 (24)	**0.010**	33	88 (27)	66 (19)	**<0.001**	0.229	0.062
Protein intake (g/day)	48	77 (20)	75 (20)	0.591	33	75 (18)	76 (18)	0.865	0.736	0.226
Cholesterol (mg/day)	48	306 (127)	301 (145)	0.858	33	308 (153)	249 (102)	**0.029**	0.948	0.268
Fiber (g/day)	48	14 (5)	14 (5)	0.641	33	14 (9)	16 (6)	0.281	0.720	0.400
Calcium (mg/day)	48	661 (217)	665 (201)	0.920	33	639 (203)	659 (229)	0.610	0.948	0.898
Magnesium (mg/day)	48	234 (57)	234 (58)	0.963	33	233 (121)	248 (73)	0.454	0.647	0.299
Sodium (mg/day)	48	2227 (1003)	2022 (756)	0.199	33	2191 (764)	2248 (776)	0.688	0.948	0.068
Potassium (mg/day)	48	2352 (480)	2430 (653)	0.442	33	2292 (795)	2540 (644)	0.090	0.718	0.107
Vegetables (g/day)	48	76 (58)	93 (77)	0.192	33	111 (107)	140 (85)	0.171	0.090	0.363
Fruits (g/day)	48	149 (126)	240 (168)	**0.001**	33	138 (155)	189 (166)	**0.027**	0.727	0.420
Dairy products (g/day)	48	333 (156)	336 (134)	0.910	33	306 (162)	339 (152)	0.239	0.452	0.658
Low-fat/skimmed dairy (g/day)	48	109 (144)	179 (157)	**0.001**	33	93 (138)	174 (174)	**0.014**	0.622	0.672
Cereals (g/day)	48	166 (72)	160 (61)	0.577	33	145 (57)	175 (71)	0.070	0.144	0.028
Whole cereals (g/day)	48	12 (23)	19 (32)	0.177	33	4 (11)	7 (15)	0.460	0.055	0.413
Nuts and legumes (g/day)	48	16 (21)	14 (21)	0.743	33	13 (17)	16 (19)	0.493	0.605	0.742
Fish and seafood (g/day)	48	41 (53)	41 (47)	0.958	33	34 (48)	30 (28)	0.739	0.535	0.976
Meat and meat products (g/day)	48	104 (88)	81 (64)	0.162	33	93 (62)	89 (57)	0.916	0.519	0.084
Sugar-sweetened beverages(g/day)	48	85 (146)	47 (81)	0.135	33	63 (92)	37 (101)	0.282	0.412	0.313
**Adherence to dietary patterns**										
KIDMED score (0–12)	45	5.7 (1.9)	8.1 (1.9)	**<0.001**	25	5.4 (2.1)	7.7 (2.0)	**<0.001**	0.552	0.652
DASH score (0–9)	48	1.3 (0.9)	1.6 (1.3)	0.182	33	1.1 (1.0)	1.9 (1.1)	**<0.001**	0.320	**0.048**
HDI score (0–7)	48	1.6 (1.1)	1.6 (1.1)	1.000	33	1.8 (1.2)	1.8 (0.9)	0.891	0.531	0.949
**Breakfast habits**										
Skipping breakfast (N, %)	48	0, 0	1, 2.1	0.100	33	1, 3	2, 6.1	0.100	0.407	0.652
BQI score (0–10)	48	3.9 (1.0)	5.2 (1.6)	**<0.001**	33	3.7 (0.9)	4.3 (1.2)	**0.025**	0.269	0.207
**Meal frequency and daily energy distribution**										
Having ≥ 4 meals/day (N, %)	48	45, 93.8	44, 91.7	0.100	33	28, 84.8	32, 97.0	0.125	0.173	0.154
Evening/morning energy intake ratio	48	0.73 (0.29)	0.61 (0.24)	**0.014**	33	0.71 (0.28)	0.69 (0.22)	0.749	0.796	0.322

KIDMED, Mediterranean Diet Quality Index for children and teenagers; DASH, Dietary Approaches to Stop Hypertension; HDI, Healthy Diet Indicator; BQI, Breakfast Quality Index. * = *p* value refers to differences between Pre- vs. Post-intervention values for either control or exercise group analyzed by Paired-samples *t* test (continues variables) and McNemar test (categorical variables). ** = *p* value refers to differences between baseline values between control and exercise group analyzed by Independent-samples *t* test (continues variables) and chi-square test (categorical variables). *** = *p* value refers to differences in changes (Post-Pre-intervention values) between the control and the exercise groups analyzed by Univariate Linear Models adjusting with sex, age and changes in energy intake as covariates; and chi square test by using pre-post changes (categorical variables).

**Table 3:** Associations of changes in dietary intake with changes in percentage hepatic fat and adiposity markers among children with overweight/obesity.

	Δ FMI (kg/m^2^)		Δ Abdominal Fat (kg)		Δ Hepatic Fat (%)	
	r	*p*	r	*p*	r	*p*
Main nutritional goals *						
Δ Energy intake (kcal/day) **	0.086	0.466	0.008	0.946	0.136	0.245
Δ Fat intake (g/day)	0.056	0.665	0.064	0.619	0.233	0.066
Δ Simple sugar (g/day)	−0.087	0.496	−0.100	0.438	0.013	0.919
Δ Fruits and vegetables (g/day)	0.136	0.288	−0.007	0.956	−0.045	0.729
Δ SSB consumption (g/day)	−0.017	0.897	−0.083	0.516	0.266	0.035
Δ Meal frequency (times/day)	−0.105	0.414	−0.081	0.528	−0.097	0.451
Dietary patterns						
Δ KIDMED score	0.004	0.976	−0.080	0.535	0.191	0.134
Δ DASH score	−0.166	0.194	−0.113	0.376	−0.137	0.285
Δ HDI score	−0.096	0.453	−0.043	0.740	0.157	0.220
Δ BQI score	0.004	0.976	0.022	0.864	−0.062	0.628

KIDMED, Mediterranean Diet Quality Index for children and teenagers; DASH, Dietary Approaches to Stop Hypertension; HDI, Healthy Diet Indicator; BQI, Breakfast Quality Index. Analyses were adjusted for sex, age and changes in height and energy intake. Δ means changes calculated as post-value subtracted by pre-value (Δ = post-pre). * Main nutritional goals of the family-based lifestyle education program. ** Adjusted for sex, age and changes in height.

## Supplementary Materials

The following are available online at https://www.mdpi.com/2072-6643/12/5/1443/s1, Figure S1: Children´s growth during the study. Weight, height and body mass index measurements at baseline, 7th and 14th weeks and at the end of the intervention (Post) in children participating in the study, Table S1: Criteria for the dietary patterns calculation, Table S2: Associations of changes in dietary habits with percent hepatic fat and adiposity markers by intervention group.
