# Supplementary material for: The Effect of a Family-Based Lifestyle Education Program on Dietary Habits, Hepatic Fat and Adiposity Markers in 8–12-Year-Old Children with Overweight/Obesity
Source: Nutrients. 2020 May 16;12(5):1443. doi: 10.3390/nu12051443 (PMC7284532; doi:10.3390/nu12051443)
Supplement: Supplementary file 1 [file nutrients-12-01443-s001.pdf]

Table S1. Criteria for the dietary patterns calculation.

Dietary Approaches to Stope Hypertension (DASH)		
**Nutrient**	**Age 8 to 10 years**	**Age 11 to 13 years**
Total fat (%E)	≤27% of energy	≤27% of energy
Saturated fat (%E)	≤6% of energy	≤6% of energy
Protein (%E)	≥18% of energy	≥18% of energy
Cholesterol (mg)	≤150 mg	≤150 mg
Fiber (g)	≥25g	≥26 g girls; ≥31 g boys
Calcium (mg)	≥1000 mg	≥1300 mg
Magnesium (mg)	≥240 mg	≥ 240 mg
Potassium (mg)	≥3800 mg	≥4500 mg
Sodium (mg)	≤2300 mg	≤2300 mg
**Healthy Diet Indicator (HDI)**		
**Components**	**0 points**	**1 point**
Saturated fatty acids (%E)	≥10	<10
Polyunsaturated fatty acids (%E)	<6 or>10	6-10
Cholesterol (mg/day)	≥300	<300
Proteins (%E)	<10 or >15	10-15
Fiber (g/day)	<25	≥25
Fruits and vegetables (g/day)	<400	≥400
Simple sugars (%)	≥10	<10
**Breakfast Quality Index (BQI)**		
**Components**	**1 points**	**0 point**
Cereals and derivate	Bread, non-sugar rich breakfast cereals	Biscuits, pastries, sugar rich breakfast cereals
Fruits and vegetables	Fresh fruit, natural fruit juices, tomato	Artificial juices, jam
Dairy products	Whole or skimmed milk, yoghurt, cheese	Dairy desserts
Food rich in simple sugars	<5% of total daily energy from simple sugars	≥5% of total daily energy from simple sugars
MUFA-rich products	Olive oil added by the consumer	Olive oil from biscuits or other fats such as butter
MUFA/SFA ratio	≥ 2/1	< 2
Energy intake	20-25% of daily energy intake from breakfast	<20% or >25% of daily energy intake from breakfast
Fruits, cereals and dairy product	To include the 3 of the components	Not to be composed of three of the components
Calcium	≥ 200mg	<200mg
Absence of butter or margarine	Not to include butter or margarine in the breakfast	To include butter or margarine in the breakfast

%E, percentage from total energy intake; MUFA, monounsaturated fatty acids; SFA, saturated fatty acids

Table S2. Associations of changes in dietary habits with percent hepatic fat and adiposity markers by intervention group.

	Control group						Exercise group					
	Δ FMI (kg/m²)		Δ Abdominal fat (kg)		Δ Hepatic fat (%)		Δ FMI (kg/m²)		Δ Abdominal fat (kg)		Δ Hepatic fat (%)	
	r	P	r	P	r	P	r	P	r	P	r	P
**Main nutritional goals ***												
Δ Energy intake (kcal/day)**	-0.001	0.997	0.071	0.652	0.001	0.993	0.380	**0.042**	0.059	0.759	0.265	0.165
Δ Fat intake (g/day)	-0.038	0.816	0.038	0.818	0.102	0.535	0.038	0.872	-0.028	0.908	0.336	0.148
Δ Simple sugar (g/day)	-0.087	0.597	-0.101	0.540	0.095	0.563	-0.022	0.925	-0.038	0.874	-0.092	0.700
Δ Fruits and vegetables (g/day)	0.209	0.202	0.036	0.826	0.028	0.866	0.016	0.948	-0.023	0.923	-0.232	0.325
Δ SSB consumption (g/day)	-0.058	0.726	-0.021	0.899	0.362	**0.024**	0.010	0.968	-0.351	0.129	0.100	0.675
Δ Meal frequency (times/day)	-0.155	0.346	-0.134	0.417	-0.102	0.536	0.075	0.753	0.079	0.740	-0.026	0.914
**Dietary patterns**												
Δ KIDMED score	0.171	0.299	0.109	0.507	0.283	0.081	-0.252	0.283	-0.375	0.103	0.110	0.645
Δ DASH score	-0.066	0.691	-0.051	0.760	0.016	0.922	-0.228	0.333	-0.191	0.420	-0.255	0.278
Δ HDI score	-0.137	0.406	-0.026	0.874	0.247	0.130	0.064	0.789	-0.018	0.940	0.155	0.514
Δ BQI score	-0.077	0.639	0.031	0.852	-0.046	0.780	-0.067	0.778	-0.020	0.935	-0.442	0.051

KIDMED, Mediterranean Diet Quality Index for children and teenagers; DASH, Dietary Approaches to Stop Hypertension; HDI, Healthy Diet Indicator; BQI, Breakfast Quality Index. Analyses were adjusted for sex, age and changes in height and energy intake. Δ means changes calculated as post-value subtracted by pre-value (Δ = post-pre). *Main nutritional goals of the family-based lifestyle education program. **Adjusted for sex, age and changes in height.

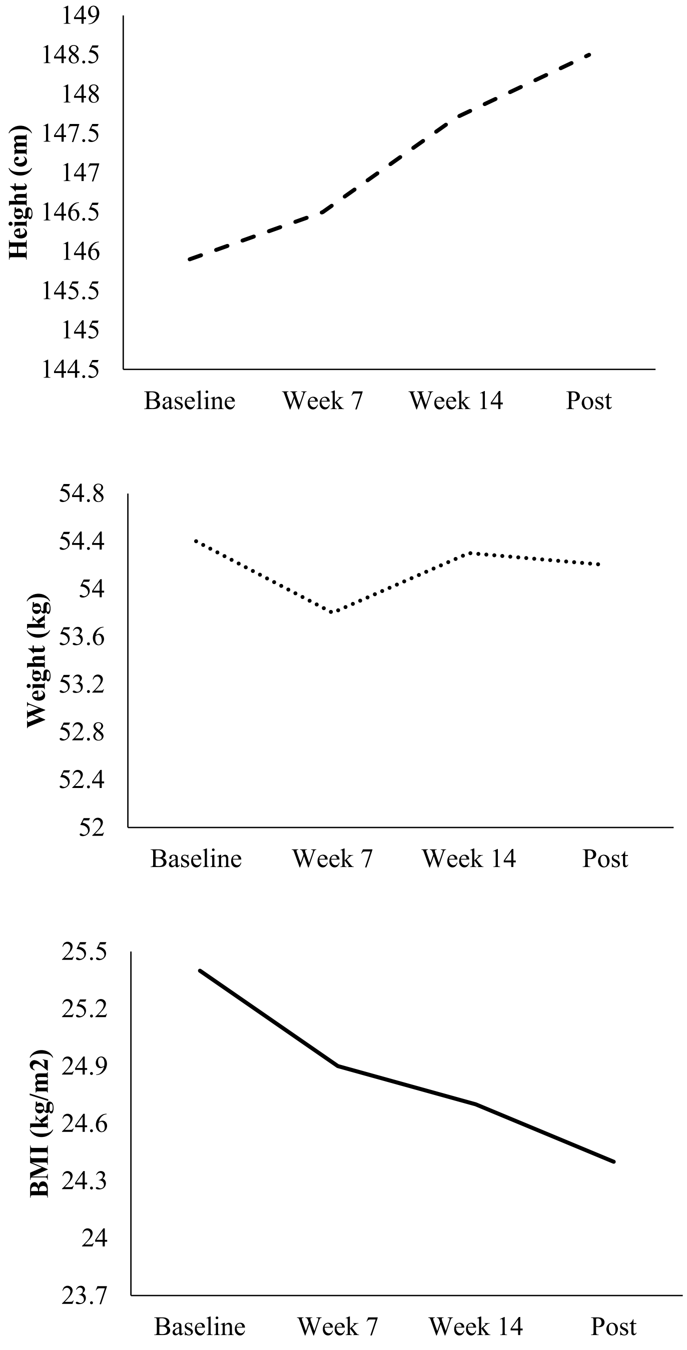

Figure S1. Children´s growth during the study. Weight, height and body mass index measurements at baseline, 7th and 14th weeks and at the end of the intervention (Post) in children participating in the study.
